# A comparative study of S/MAR prediction tools

**DOI:** 10.1186/1471-2105-8-71

**Published:** 2007-03-02

**Authors:** Kenneth Evans, Sascha Ott, Annika Hansen, Georgy Koentges, Lorenz Wernisch

**Affiliations:** 1 School of Crystallography, Birkbeck College, Malet Street, London, WC1E 7HX, UK; 2 Functional Genomics Laboratory, Wolfson Institute for Biomedical Research, University College London, The Cruciform Building, Gower Street, London WC1E 6AU, UK

## Abstract

**Background:**

S/MARs are regions of the DNA that are attached to the nuclear matrix. These regions are known to affect substantially the expression of genes. The computer prediction of S/MARs is a highly significant task which could contribute to our understanding of chromatin organisation in eukaryotic cells, the number and distribution of boundary elements, and the understanding of gene regulation in eukaryotic cells. However, while a number of S/MAR predictors have been proposed, their accuracy has so far not come under scrutiny.

**Results:**

We have selected S/MARs with sufficient experimental evidence and used these to evaluate existing methods of S/MAR prediction. Our main results are: 1.) all existing methods have little predictive power, 2.) a simple rule based on AT-percentage is generally competitive with other methods, 3.) in practice, the different methods will usually identify different sub-sequences as S/MARs, 4.) more research on the H-Rule would be valuable.

**Conclusion:**

A new insight is needed to design a method which will predict S/MARs well. Our data, including the control data, has been deposited as additional material and this may help later researchers test new predictors.

## Background

In the nucleus of eukaryotic cells specific regions of the DNA are attached to the nuclear matrix. These regions are called matrix attachment regions (or scaffold attachment regions, abbreviated as S/MARs). It is thought that there are tens of thousands of S/MARs in the genome of higher eukaryotes [1], which assigns a major role in the organisation of the chromatin within the nucleus to the S/MARs. There is a category of S/MARs that function as boundary elements when they separate a gene from other genes' regulatory modules [2-5]. S/MARs can activate enhancer regions [6], and determine which one of a class of genes to transcribe [7]. They also have a strong effect on the level of expression of transgenes [8,9]. Therefore, S/MARs are of intrinsic interest for the understanding of gene regulation in eukaryotic cells.

Reliable predictions of S/MARs by computer would be very valuable, as they would facilitate the design of experiments and improve our understanding of regulatory mechanisms. In genome-wide applications, such methods could allow insights into the number of S/MARs [10], their distribution in the genome, their position relative to genes, and the functional classifications of S/MARs. However, while a number of methods have been proposed, their predictive power has so far not been put under detailed scrutiny and is therefore uncertain. There has also been very little analysis of the respective strengths and weaknesses of the proposed methods which could facilitate the design of better methods.

A considerable amount of research has been focused on computational predictions of the position of S/MARs and several methods of S/MAR prediction have been proposed. The MAR-Finder method scores sub-sequences of DNA by the abundance of DNA-motifs thought to be correlated with S/MARs [11]. Particular motifs have been suggested by experimental groups: the recognition signature (MRS) consisting of two consensus sequences [12] and a "consensus" sequence by Wang *et al. *[13]. It has also been found that a long run of bases that do not contain a G binds to the matrix [14] and this is the basis of the H-Rule [15]. Two methods which have attempted to learn motifs from a training set are SMARTest [10] and ChrClass [16], the latter also attempting to classify S/MARs.

It has been recognised that S/MARs are often AT-rich and this has lead to the idea that strand separation (or at least the potential for strand separation) is important for S/MAR binding. The program Thermodyn [17] makes a simple calculation of the free energy of strand separation and this program has received a fleeting mention in the S/MAR literature [18] where its results correlated with the S/MARs observed in that experiment.

SIDD (stress-induced duplex destabilisation) is a more complicated calculation of the potential of DNA strands to separate in a given region [19]. This calculation takes into account the torsional stress on the DNA and uses a thermodynamic model of energy states. A long series of papers [2,19-21] has drawn attention to correlations between S/MARs and SIDD results in particular situations and experiments. Other authors such as Krawetz *et al. *[22] have included SIDD in their list of possible tools for finding S/MARs. However, the latest thinking of the SIDD team [21] is that SIDD calculations do not [yet] form the basis of an S/MAR predictor for wild type S/MARs in genomic DNA. In view of the obvious interest in this approach, the following analyses include our own interpretation of the method which we call "duplex destabilisation".

It is not surprising that the original authors make encouraging statements about their own methods but a number of authors have praised competitor methods, for example:- The authors of SMARTest say MAR-Finder gives 80% precision and 32% sensitivity [10]. Rogozin *et al. *[23] say that both MAR-Finder and ChrClass (their own method) can be recommended for analysing eukaryotic genomes even if caution must be exercised. A paper from the SIDD team [20] compared MAR-Finder, SMARTest and SIDD on one sequence and said "we have applied all three algorithms in parallel and found a reassuring amount of coincidence for a thoroughly studied example." The experimental paper by Purbowasito *et al. *[24] complimented the good performance of ChrClass with 85% sensitivity and 50% precision. Another experimental paper [25] found the MRS signature to be a good indicator.

There have however been a handful of negative results in the literature. The experimental paper by Ostermeier *et al. *[26] failed to find any correlation with predictive methods. Liebich *et al. *[27] suggested that many motifs associated with S/MARs were merely a consequence of the fact that many S/MARs were AT-rich. Although Purbowasito *et al. *[24] complimented the performance of ChrClass, other methods were not found to be so successful. Krawetz *et al. *[22] give a small tutorial example on the use of computer methods where it is notable that the methods give different results and the advice is "to compare the results obtained from several different algorithms".

On balance the tone of the literature is very positive, even if this does not reflect the views of many workers in the field, especially for the prediction of AT-poor S/MARs. The situation calls for a comprehensive statistical evaluation especially since these methods are used by biologists to plan their experiments. Our analysis uses relatively large datasets and in particular measures performance against negative control sets.

We have put together a positive test set of experimentally verified S/MARs of known position within the mouse or human genome as well as negative test sets and applied all the methods mentioned above to this data. Our evaluation reveals that these methods have little predictive power. Moreover, we show that a simple rule based on AT-percentage generally achieves the same level of accuracy as the other methods. It is well known that many S/MARs are AT-rich but it is still a surprise that such a simple method compares well with the current state of the art.

A point of practical importance to the user is that the methods will largely predict different sub-sequences. This result might be exploited to design a stronger method for S/MAR prediction, but we believe further insights based on the biological mechanisms involved will be needed.

Although none of the methods analysed could serve as a practical prediction tool, our analyses suggest several reasons for thinking further research on the H-Rule would be valuable.

Extensive additional material has been deposited [see Additional file 1].

## Results

**Figure 1:**
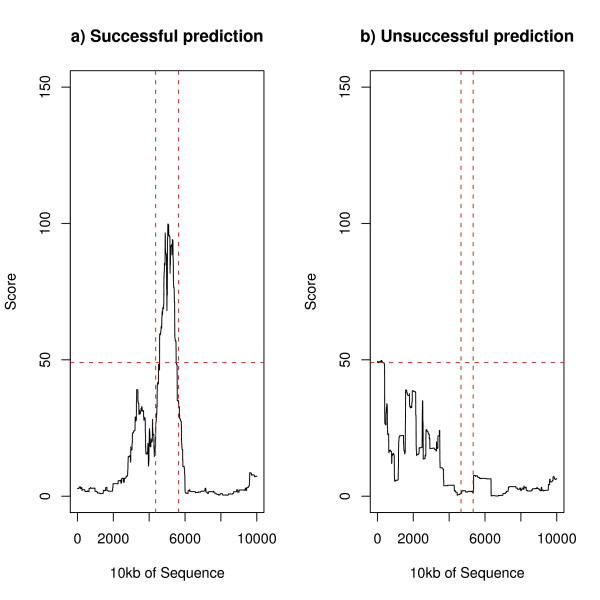
An example of a successful and an unsuccessful prediction of MAR-Finder (rules 1–6)–sequences surrounding SM217 and SM418 respectively.

**Figure 2:**
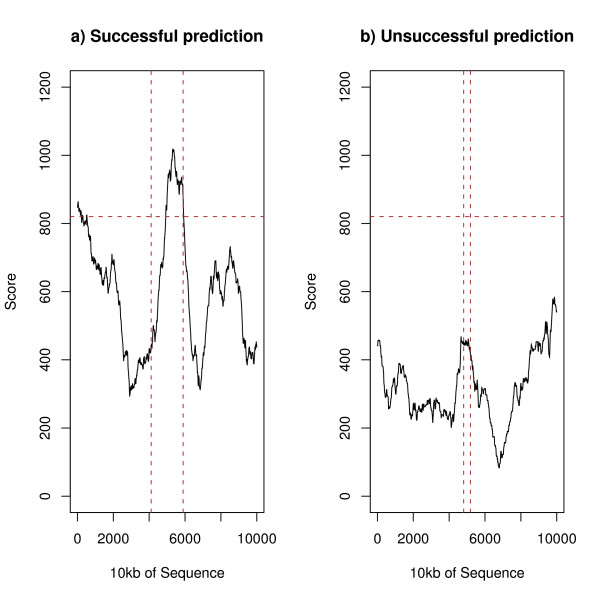
An example of a successful and an unsuccessful prediction of the H-Rule–sequences surrounding SM003 and SM015 respectively.

We have applied each of the methods to each sequence in the positive and control test sets as explained in the Methods section–Figures 1 and 2 give examples of successful and unsuccessful predictions of two methods for sequences in the positive dataset. The vertical lines give the extent of the S/MAR and the horizontal line a representative threshold (as used in Table 1 below).

**Table 1:** Percentage of S/MARs and pseudo-S/MARs predicted by each method by type of sequence

	Positive	Background	Negative	Coding	*E. coli*
MAR-Finder (rules 1–6)	18.2%	9.4%	0.0%	0.0%	4.5%
MAR-Finder (rules 1–5)	10.3%	9.4%	0.0%	0.0%	1.5%
Duplex destabilisation	13.3%	9.4%	16.4%	15.2%	8.5%
ChrClass	13.9%	10.9%	1.8%	0.6%	0.9%
H-Rule	17.6%	9.7%	0.0%	0.0%	0.0%
Thermodyn	18.8%	9.4%	0.0%	0.0%	0.0%
AT-method	15.2%	9.4%	0.0%	0.0%	0.0%

**Figure 3:**
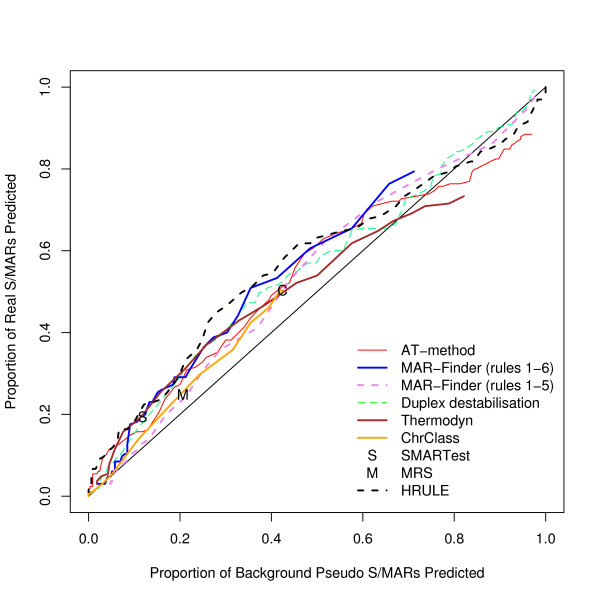
ROC curve for the positive dataset versus background sequences.

MAR-Finder (rules 1–6) includes the "AT-richness rules", and MAR-Finder (rules 1–5) excludes this rule. Two or more hits for the same S/MAR counted as one. This Table shows that there is little difference between the results for the positive and background datasets. The ranking of the methods depends on the chosen background discovery rate (see Figure 3).

We have found the proportion of true positives and false positives for each method. The trade-off between finding more true positives at the expense of finding more false positives is given by the "Receiver Operator Characteristic" (ROC) and Figure 3 gives a ROC curve where the true sequences are taken from our dataset of positive sequences and the false sequences are taken from our preferred control dataset, the background dataset. This graph shows that the discrimination of all the methods is very low. The curves barely rise above the diagonal which represents a random classifier. Table 1 gives results for methods with a variable threshold where the threshold has been set to give a background "discovery rate" around 10%, and Table 2 for the other two methods. These predictions are of questionable practical use.

**Table 2:** Percentage of S/MARs and pseudo-S/MARs predicted by each method by type of sequence

	Positive	Background	Negative	Coding	*E. coli*
MRS	24.8%	20.6%	7.9%	1.2%	7.0%
SMARTest	19.4%	11.8%	0.0%	0.3%	0.6%

Two or more hits for the same S/MAR counted as one.

Results have also been obtained for a number of different combinations of the data (including the parts of the positive dataset obtained from the SMARt DB and Purbowasito sources separately)–but no method can be said to be good in any of the conditions tested.

**Figure 4:**
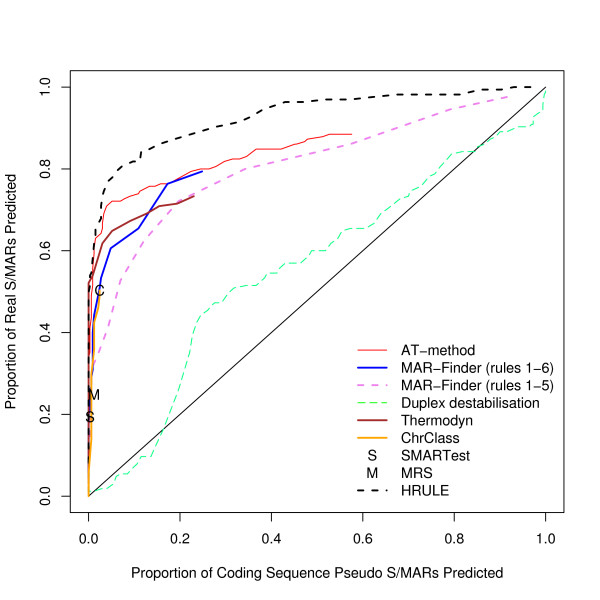
ROC curve for the positive dataset versus coding sequences.

**Figure 5:**
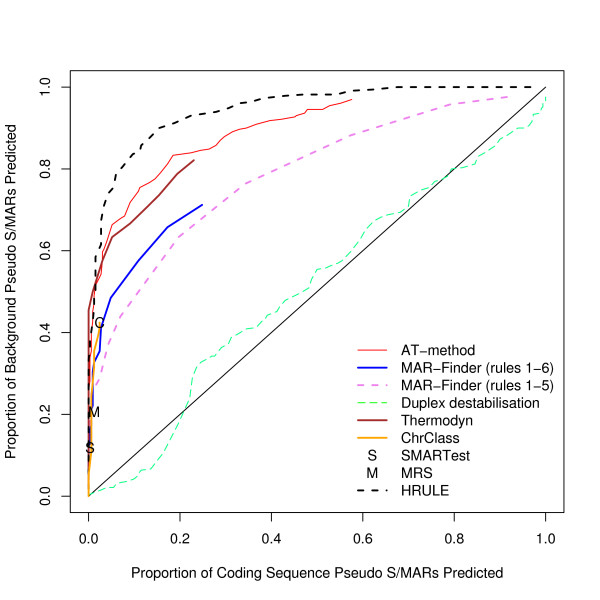
ROC curve for the background dataset (that is randomly selected real mouse DNA) versus coding sequences.

Analyses using three other control sets have also been performed. A complete set of graphs for these other control data is available [see Additional file 2]. The results for these three datasets are very similar to each other and we have chosen the coding dataset used in Figure 4 to illustrate these other results. If this result is taken at face value then the H-Rule would be the best and the AT-method next best. However, we doubt the value of this result: Figure 5 shows how effective these predictors are in distinguishing background mouse/human DNA from coding DNA and this graph shows that the predictors are doing little more than this.

**Figure 6:**
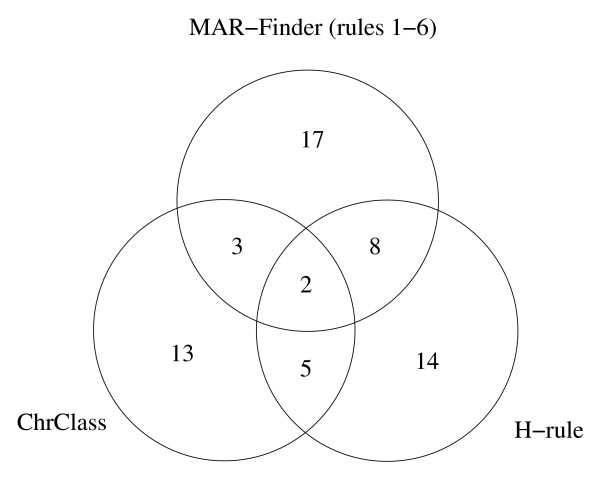
**A Venn diagram showing the number of S/MARs found at Table 1 thresholds**. This Venn diagram shows the number of S/MARs found by various combinations of three methods at the thresholds used for Table 1. 103 S/MARs were not found by any of these three methods at these thresholds.

In practice the methods will find different sequences: this can be seen from Figure 6 where the Venn diagram shows the overlap of the sets of positive sequences predicted to contain S/MARs by three methods, at the thresholds used for Table 1: ChrClass, the H-Rule and MAR-Finder (rules 1–6)–the MAR-Finder rules are explained in reference [11] and summarised in Table 3 below. The additional material contains a table showing which S/MARs were found [see Additional files 3 and 4]. Results for other combinations of methods or thresholds will lead to the same conclusion.

**Table 3:** Summary of the MAR-Finder Rules

Rule Number	Purpose of Rule
1	Origin of replication
2	TG-richness
3	Curved DNA
4	Kinked DNA
5	Topoisomerase II cleavage sites
6	AT-richness

7	Consensus motif
8	ATC rule

Each rule is based on a set of motifs thought to be related to a biological feature which itself is likely to be indicative of an S/MAR. In standard use, rules 1–6 are used.

**Figure 7:**
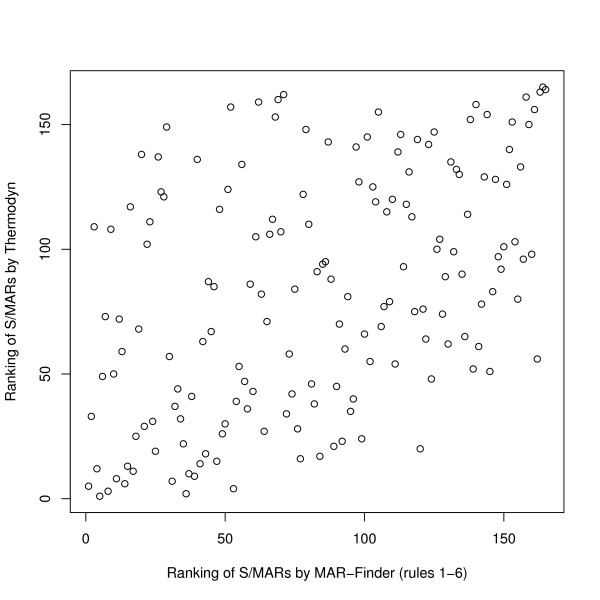
**Comparison of Thermodyn and MAR-Finder (rules 1–6)**. Shown are the ranks of each S/MAR in the positive test set according to the threshold at which it is detected. The higher the correlation the more the methods agree on the order in which the S/MARs are detected using varying thresholds. S/MARs with the strongest signals are top right. The correlation is 0.46.

However, because of the small sample size, we prefer the following presentation. Each method ranks each S/MAR in the positive test set according to the threshold at which it is detected: if two methods are finding the same S/MARs then a plot of the ranks of the two methods against each other will be a straight line. Figure 7 shows an example: it compares Thermodyn with MAR-Finder (rules 1–6)–the high ranks (top right of the plot) are the points which the methods predict as the most likely S/MARs. The correlation of the points in this plot is 0.46. Other pairs of methods give similar results.

**Figure 8:**
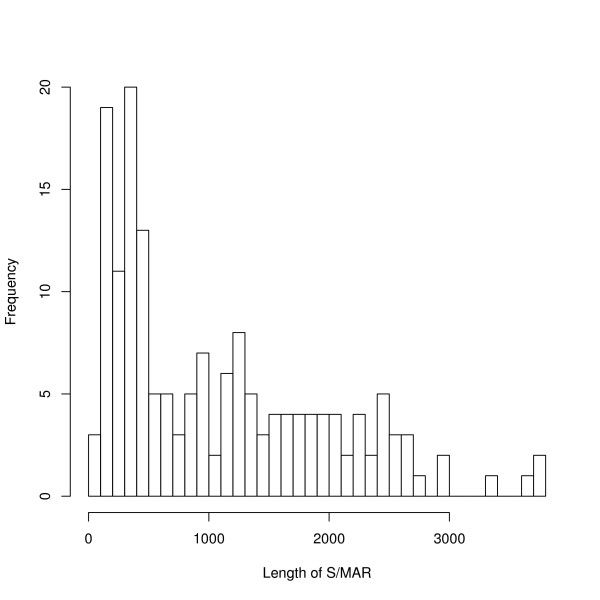
Histogram of length of S/MARs.

**Figure 9:**
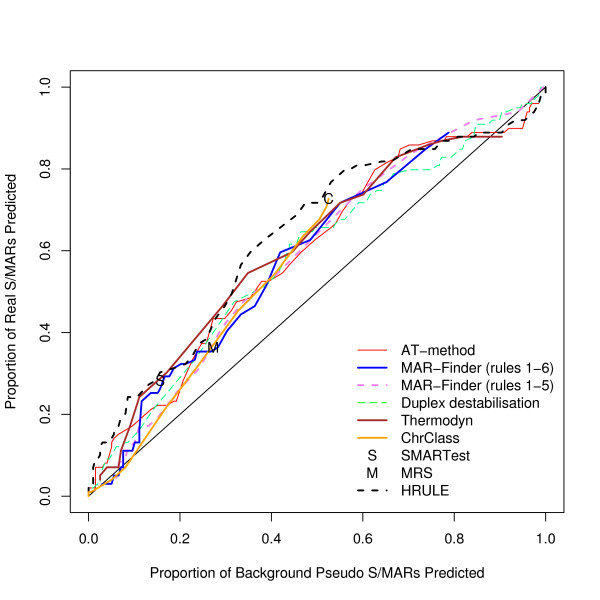
ROC curve for long positive S/MARs versus background sequences.

The length of the S/MAR, as defined in the database, is a feature which makes S/MARs easier to predict. Figure 8 gives a histogram of the lengths of the S/MARs in the positive test set. These lengths have a median of 850 bases and a mean of 1092 bases. However, the median lengths of S/MARs identified for the thresholds in Tables 1 and 2 are much longer: these medians range from 1274 to 1966 which are the values for the AT-method and ChrClass respectively. This is not a consequence of the fact that a random sequence is more likely to be found if it is longer: Figure 9 shows the ROC curve for S/MARs of 500 bases or longer in the positive dataset and both the discovery rate and the discrimination improves.

**Figure 10:**
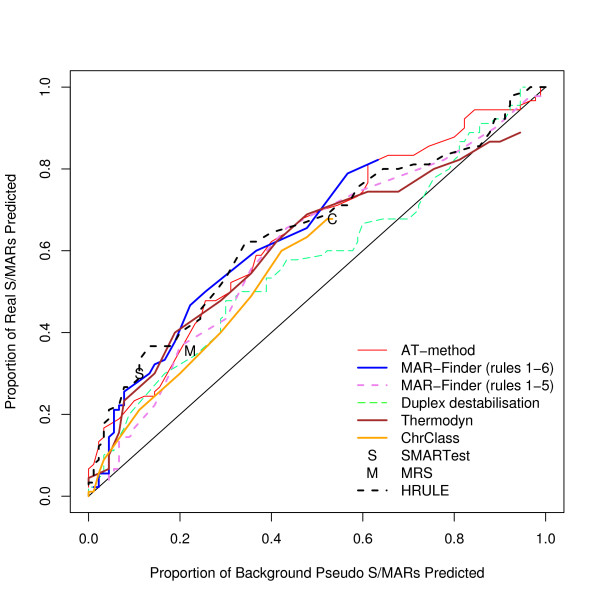
ROC curve for AT-rich 10 kb regions.

**Figure 11:**
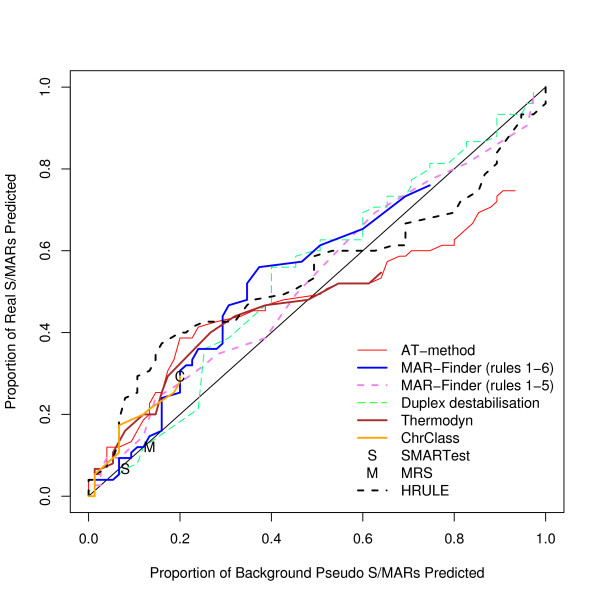
ROC curve for AT-poor 10 kb regions.

We have considered how the AT% of the 10 kb region affects the results: we have used a median value of 58% for the AT% as the cut off and divided positive and background datasets into AT-rich and AT-poor. The results for finding S/MARs in AT-rich regions compared with the results for pseudo S/MARs in AT-rich regions are given in Figure 10 and the corresponding results for AT-poor regions in Figure 11. The general result is that all methods perform slightly better in AT-rich regions than in AT-poor regions. These results do not support the suggestion that rule 6 of MAR-Finder should be used for AT-rich regions and not for AT-poor regions. The H-rule is best or near the best in both circumstances. However none of the results are indicative of a good predictor.

**Figure 12:**
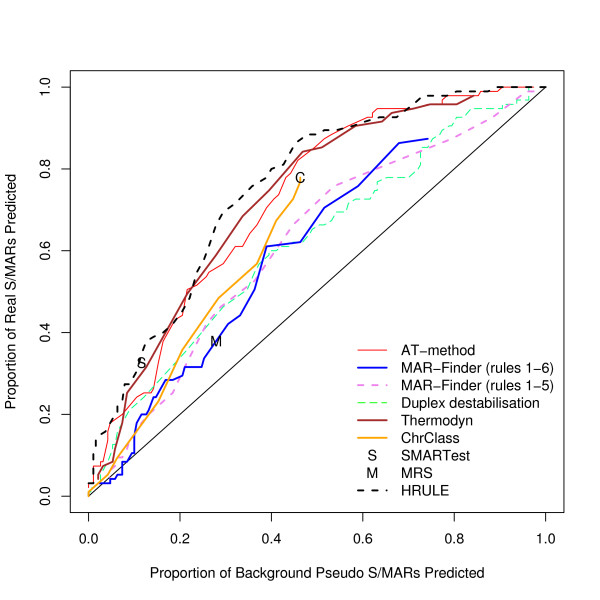
ROC curve for finding S/MARs that are AT-rich.

**Figure 13:**
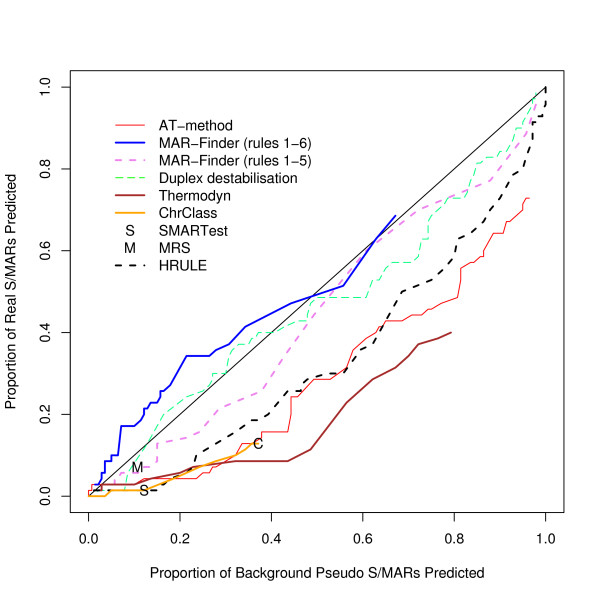
ROC curve for finding S/MARs that are AT-poor.

We have also looked at the AT% of the S/MAR itself to see how this affected the success of the predictor: of course the AT% of the S/MAR is not known until it is found. For this comparison no attempt was made to control for the AT% within the background S/MAR. Figure 12 shows that AT-rich S/MARs are easier to find–a result in line with expectations. The H-Rule, Thermodyn, the AT-method and SMARTest are the best methods in this analysis. However, the results for AT-poor S/MARs in Figure 13 are worse than random predictions: only MAR-Finder (rules 1–6) remains above the diagonal. Given that all the methods are looking for some definition of AT-richness this result is understandable even if Figure 13 exposes the problems of the methods.

**Figure 14:**
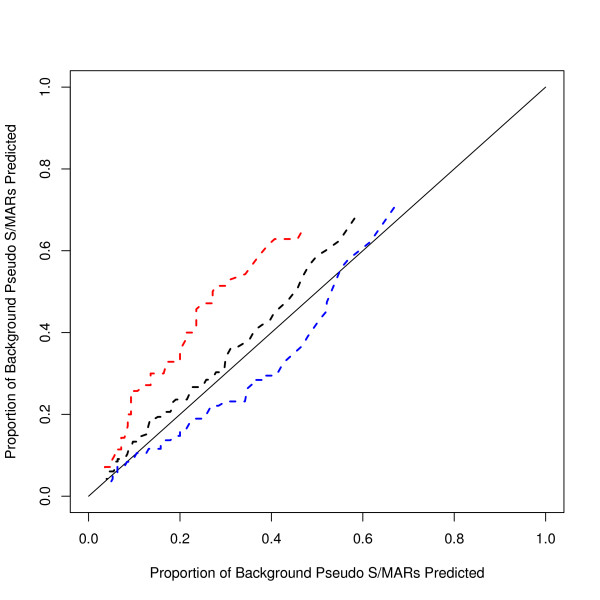
**ROC curve for the H'-Rule for three divisions of the dataset**. This Figure compares the positive dataset with the background dataset. a) black (middle curve) using all the positive dataset; b) red (top curve) AT-poor S/MARs; c) blue (bottom curve) AT-rich S/MARs. Note the contrast with the H-rule in Figures 12 and 13.

**Figure 15:**
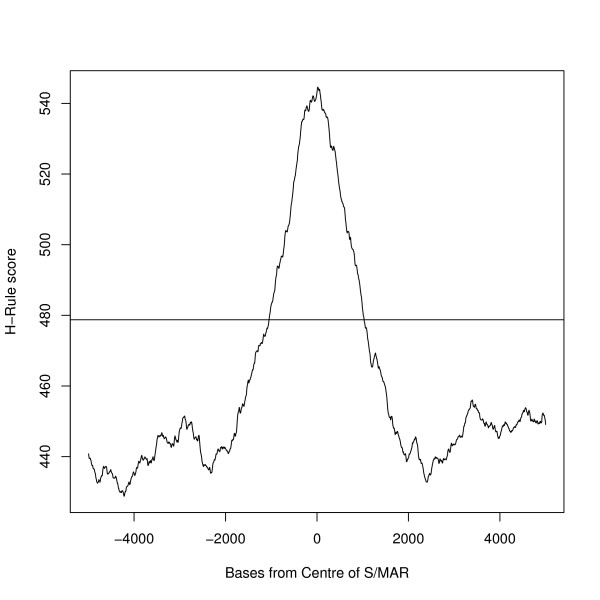
**H-Rule measure**. This Figure shows the average of real S/MAR sequences (line with peak) compared with the average background level (the horizontal line).

MAR-Finder uses the base frequencies of the local sequence in its calculations and this may be relevant to its performance in Figure 13. Conversely, as explained in the Methods section the H-rule is based on an absolute count of motif occurrences. We therefore tested if the MAR-Finder interpretation of the H-Rule would be better–Figure 14 shows the results for this version (here called the H'-rule). We see that judged on the total dataset it gives a poor result even by the standards of the methods available. However, for the *AT-poor *S/MARs it gives surprisingly good results, even if for *AT-rich *S/MARs the results fall below the diagonal. Some insight into this result can be gained from Figure 15 which shows the average value of the absolute H-Rule score by distance from the centre of the S/MAR: also shown is the average value of the background dataset. The S/MAR average shows a peak above the background but away from the S/MAR this average value falls below the background average. The S/MAR average in fact remains below the background average for a few tens of kilobases each side of the centre of the S/MAR (data not shown).

**Figure 16:**
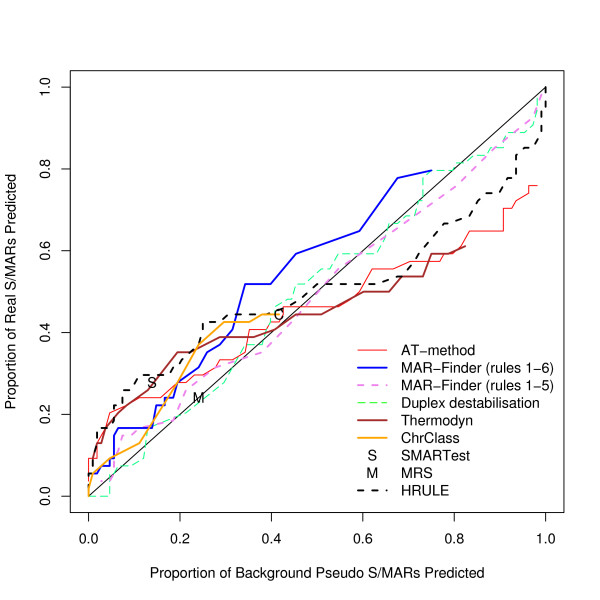
ROC curve for the positive with-LIS data versus background sequences.

**Figure 17:**
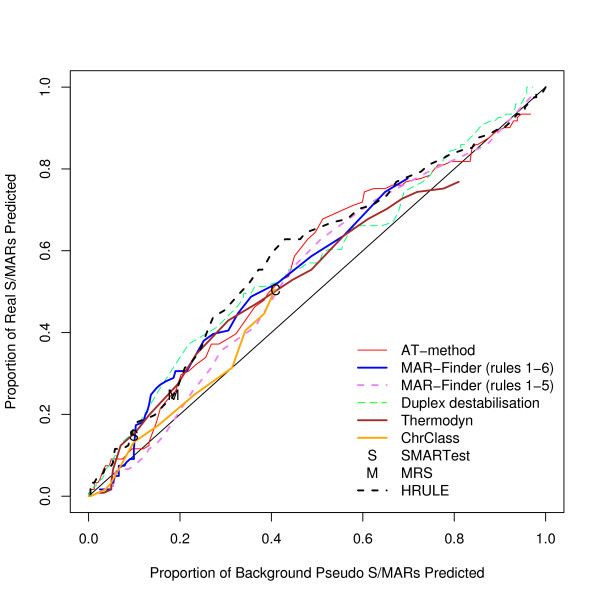
ROC curve for the positive non-LIS data versus background sequences.

To see the effect of using LIS–see the discussion of experimental protocols in the Methods section–we give Figures 16 and 17 which show the results for the S/MARs confirmed with and without LIS. There are some indications that some methods differ in their ability to find these two types of S/MARs: for example SMARTest, the H-Rule and Thermodyn seem to be better at finding LIS verified S/MARs. Although we were unable to find any combination of results which was statistically significant in the dataset, it is possible that this is because the dataset is too small. We also considered if the *in vitro *verified S/MARs gave different results to the *in vivo *verified S/MARs, but could not find such an effect. For both comparisons it is necessary to control for the different lengths of the positive S/MARs in the subsets of the data.

## Discussion

We have evaluated the predictive power of the available methods for S/MAR prediction on positive and background test sets using straightforward analytical techniques. The results lead us to four main conclusions: the methods analysed have little predictive power; a simple rule based on AT-percentage does just as well especially at low false discovery rates; in practice, the different methods will identify different sub-sequences; several aspects of the H-Rule have been observed which deserve further investigation.

These results are different from those claimed by the original authors and this needs some explanation. We think that the main reason for these conclusions is that much previous analysis did not include a comparison against negative/background test sets and in particular a control based on the length of the putative S/MAR. Several of the original studies are based on only a handful of positive test sequences. Their results cannot be generalised to these larger human/mouse test sets. We also question if some of the analyses in the literature have not been biased by a concentration on one or two of the best cases effectively using very stringent thresholds. As an aid for future work we have included the 10 kb sequences for both the positive and control sequences in the additional material [see Additional files 5, 6, 7, 8, 9].

We have explored changes in the details of the analyses. For example we tried doubling/halving the window sizes. For MAR-Finder we have tried other combinations of rules, alternatives in defining the base probabilities and how the signals for the two strands are combined. The thrust of our conclusions is unchanged. We are therefore confident that our conclusions are robust against technical changes and definitions. The Purbowasito data [24] formed about a third of our dataset. In case this affected the results, we repeated our analyses without this data and our conclusions are unaffected.

While all methods have little predictive power, some comments on individual methods can be made. In general MAR-Finder performs better when rules 1–6 are used than when only rules 1–5 are used. This is true for regions which are AT-rich as well as those that are AT-poor. Exploratory analyses suggest that MAR-Finder might perform better when the base frequencies are defined from a very long sequence which contain the candidate S/MAR(s), but this alternative does not turn the method into a strong predictor. The ChrClass method does not come with strong claims from its authors but Purbowasito *et al. *[24] found good performance for S/MARs in one megabase of DNA sequence. For the same data we find that it detects a high proportion of true S/MARs but also has a high false positive rate: for the other S/MARs in our dataset it does not perform well (details not shown). We find the MRS signature to have very moderate predictive power–less than expected [12]. The SMARTest method tends to find a smaller proportion of real (and pseudo) S/MARs than either the MRS or ChrClass methods. We also find its performance comparable to MAR-Finder at comparable levels of sensitivity. Thermodyn turns out to be as good as some of the more established methods.

The H-Rule may not be of practical use but it is the best predictor of those examined. Figures 14 and 15 show aspects of this idea which might be exploited to improve the measure. Figure 15 and the fact that the S/MAR average remains below the background average for tens of kilobases from the S/MAR centre invites interpretation. A typical S/MAR may be in a large distinctive region which presumably contains more than one S/MAR. Regions between the S/MARs (or S/MAR-clusters) may be under selective pressure not to bind to the matrix to allow for proper looping of DNA. Therefore, one way to improve S/MAR prediction may be to incorporate features of the neighbouring, non-binding regions into the model.

In framing the duplex destabilisation method, we have followed the ideas and calculational technique of the SIDD calculations [19]. We have tried variations in the method, e.g. of window sizes and the use of the p-graph instead of the G-graph and have found that they all lead to the same conclusions. We have also experimented with alternatives such as area measures (the area of graph under a given threshold) and length measures (the number of bases for which a graph falls below the threshold). For some area measures there was a small improvement in predictive power but nothing to alter the main conclusions. We did not try the complexities of the method described in general terms in [21] but we see no reason to dispute their conclusion that a SIDD predictor of wild type S/MARs in genomic DNA has not yet been developed.

It is of course possible that these principles might be used successfully but we foresee difficulties and do not share the hopes expressed in [21]. The purpose of SIDD calculations is to calculate where torsional stress causes DNA to separate. It is still not obvious that either torsional stress or strand separation is relevant to S/MAR binding. *In vivo *the salt concentration is comparatively high and DNA would relieve torsional stress by twisting about itself rather than melting [28]. The presence of nucleosomes also relieves torsional stress. Several S/MAR proteins bind to double stranded DNA and SATB1 in particular does not bind to single stranded DNA [14]. The main problem that we have found is that for any sequence the duplex destabilisation method searches for the weakest point. There is therefore a tendency for the duplex destabilisation method to predict exactly one S/MAR in any sequence of any length. To some extent this is avoided by the use of the G-graph rather than the probability p-graph–but it remains a difficulty of the method. This explains its poor performance on the coding, negative and *E. coli *test sets. It also explains the purpose behind the procedure described in [21] of obtaining a standardised measure for a sub-sequence by splicing it into a standard plasmid.

Our use of AT-percentage as a prediction rule is *not *intended to suggest that AT-richness of (many) S/MARs is a new result. On the contrary, most authors who write on the subject explain that many S/MARs are known to have a high correlation with AT-rich regions or have runs of As, but the situation is more complicated. The current methods of S/MAR prediction have a correlation with AT-richness built in: MAR-Finder has several rules correlated with AT-content and the duplex destabilisation calculations are heavily influenced by AT-content. Indeed as noted below when rule 6, "AT-richness", is included in MAR-Finder it dominates the other rules. However, we do find it striking that our simple AT-percentage-rule is competitive with published methods.

One notorious problem for research in this area is the choice of a control set. There are a number of features of the genome which have been annotated–for example transcription start sites–but it does not follow that these regions are free of S/MARs. On the contrary these may be regions where S/MARs are to be found. The approach that we prefer is to use real DNA sequences chosen randomly from the mouse genome. The disadvantage is that the real proportion of S/MARs within this test set is unknown. However, if a predictor cannot make a clear distinction between the real S/MARs and the background set it must mean that either the predictor is very poor or that the definition of an S/MAR is nearly meaningless–a random piece of DNA is just as good. We have constructed three other control datasets: the coding, the negative and the *E. coli *dataset. The advantage of these three sets is that it is almost certain that there are no S/MARs in these datasets: both the function and sequence of coding sequences make them unlikely to contain S/MARs; the negative dataset consists of an entirely artificial sequence; and *E. coli *has no nucleus which implies that no specialised S/MARs can have evolved. The disadvantage of these datasets is that they can easily be distinguished by statistical methods from mouse/human sequences and if the evaluation is set up in this form it is not clear whether a successful measure is doing anything more than distinguishing the type of sequence rather than finding S/MARs. Our reading of Figures 3, 4, 5 is that this is all these measures are doing. However, if these control sets are regarded as giving a proper evaluation then the H-Rule and the AT-method are superior to the other methods. It is also true that if one used a threshold which found only a fifth of real S/MARs then most of the methods would find a negligible number of S/MARs in these three control sets. However, on the same basis, the same threshold would identify at least a tenth of random sequences of mouse DNA as containing an S/MAR.

Users will also need to bear in mind that different methods will predict different sub-sequences as S/MARs. At one level this is obvious in that the default parameters of the different methods operate at regions along the ROC curve. It is also a corollary of the with-LIS/non-LIS results (Figures 16 and 17). Other evidence that the methods will in practice identify different sequences was given in Figures 6 and 7.

A number of roles for S/MARs have been proposed in the literature [29], and this suggests it might be possible to predict the function or type of an S/MAR as well as its presence. However, there is not yet sufficient data for such an analysis. The ChrClass method [16] is an attempt at this, but the authors themselves stress the difficulties of their analysis. We examined the notes in SMARt DB and it is possible that the different methods are in fact predicting S/MARs with different features, e.g. duplex destabilisation for S/MARs with bent DNA and MAR-Finder with clusters of motifs, but with the current limited data we can only leave this as a question for further work. The analysis splitting the data by LIS and non-LIS S/MARs fell short of giving statistical significance, but it would be interesting to see if a larger dataset could settle the question of whether some methods were better at finding LIS confirmed S/MARs.

Using background sequences allows one to see how many hits a method will generate on average in a length of sequence. Unfortunately the poor predictive power of the methods means that we cannot go on to estimate the number of S/MARs in the genome, and we have therefore not reported these results.

However, two points may be made. Firstly, as could be deduced from the Results section, there are many hits in the background sequences. Secondly, sequences containing a known S/MAR contain more hits than background sequences do–suggesting that S/MARs come in clusters. This possibility is not unexpected given the work of [7,24] and [25].

**Figure 18:**
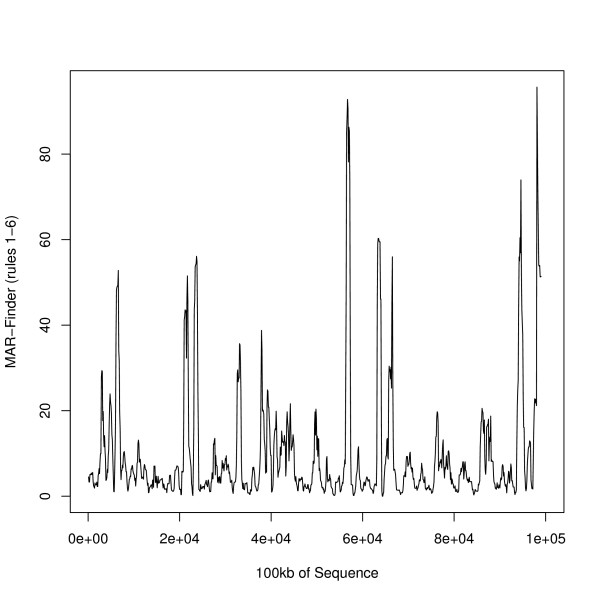
An Example Measure over an Example Sequence–sequence surrounding SM003.

It is also revealing to examine individual plots of 100 kb sequences. Figure 18 gives an example of the results for one sequence of 100 kb: this happens to be of the MAR-Finder (rules 1–6) measure but similar examples could be given for any of the measures. The known S/MAR is of length 1783 bases in the centre of the sequence where there is a small local peak whose height is lower than the many other peaks in the figure. One possibility is that for this sequence this method simply gives the wrong results. Another view is that the rule gives a near miss or there are several S/MARs in the region. This figure is typical of dozens that we have examined. The implication is that S/MARs may come in clusters which are spread over many kilobases and that the existing methods do not get to the root of the matter.

## Conclusion

We have been invited to comment on where progress might be made in this field. We do not share the view that S/MARs are no more than an experimental artefact but we do suspect that the term is too broad and useful categories need to be identified either by function or more probably by the protein (or protein family) that binds to the S/MAR. We also expect that experiments of wild type S/MARs will be more useful than *in vitro *ones. A corrollary of S/MARs coming in clusters is that a biologically relevant feature might be a long region (say 100 kb) that contains a cluster of S/MARs. Support for this idea comes from the behaviour of the H-Rule measure which takes several 10 kb to return to the background level. If the biology is to be explained in terms of strand separation then the *in vivo *mechanisms need to be better understood. On present evidence it looks more likely that the H-Rule is closer to the biological mechanisms and that progress may be made by a better mathematical formulation of the H-rule.

It appears that existing methods can pick out a few extreme candidates for S/MARs and that these can be expected to be true positives. However, a method which identifies S/MARs with good precision is still needed. It is clear that a new insight is needed: perhaps then we will be able to identify some sequences as forming–for example–insulators between genes and others as framing regulatory cassettes.

## Methods

### S/MAR Predictors

#### MAR-Finder

The MAR-Finder method [11] uses a set of DNA-sequence motifs known to be abundant in S/MARs in order to predict S/MARs. In the MAR-Finder calculations these motifs are grouped into "rules" according to the reason the motif was included: see Table 3. As noted below, the authors are not entirely prescriptive as to which of these rules are to be used.

In a window of fixed length the number of occurrences of each motif is determined and compared to the expected number of occurrences in a random DNA sequence of the same length as the window. Using a Poisson distribution these values are turned into a score for that window. The average of the score for the positive strand and the negative strand is then computed and called the *MAR-potential*. This step is repeated for each window along the sequence under consideration and those windows that have a MAR-potential above a given threshold are predicted to contain an S/MAR. In the original method the MAR-potential is scaled so that the maximum value for the sequence under consideration is 1.0. We give results by Frisch *et al. *[10] for the power of this method in the subsection describing SMARTest.

Both the original MAR-Finder method [11] and its present incarnation on the MAR-Wiz website [30] leave it open to the user to specify several steps in the analysis–in particular, which motifs to include and how the final scaling is done. An example of this ambiguity is that rule 6–the "AT-richness rule"–is used in some applications for the detection of AT-rich S/MARs, but not for the detection of AT-poor S/MARs [10,11,16]. We have evaluated the contribution of this motif to the MAR-potential and found that it dominates the other motifs, if included. The MAR-Wiz website allows two rules to be used which were not discussed in their original paper–a "consensus sequence" and the H-rule. We discuss these separately below. The default method of normalising by the maximum potential in the sequence, as described above, is statistically unstable, and the authors suggest that the user clips the highest peaks in the MAR-potential if this should seem appropriate. There is also the ambiguity of how to define the frequencies of the bases: the default is to use the frequencies in the sequence being analysed–however long that might be.

We have coded the method as described in [11]. We have deduced that the published specification is incomplete but on making the appropriate changes we get very close agreement with the graphs in the original paper and from the website. The changes refer to the motifs listed in Figure 1 of [11]. Motif 7 should read *A*_4_*n*_7_*A*_3_*n*_7_*A*_4 _and motif 8 similarly. Although not stated, it also appears that motifs 16 and 17 should be allowed to match with one and two mismatches respectively, and the probability of the motifs occurring should be adjusted accordingly.

We have used the default window of 1000 bases and tried both alternatives of including and excluding the "AT-richness rule". These are referred to as "MAR-Finder (rules 1–6)" and "MAR-Finder (rules 1–5)". Base frequencies have been calculated from the 10 kb sequence containing the S/MAR. Instead of the arbitrary scaling method we have left the MAR-potential in absolute units and tried various thresholds.

#### Consensus sequence

The "consensus sequence" is "TCTTTAATTTCTAATATATTTAGAA" and is a SATB1 recognition sequence derived from the S/MAR downstream of the mouse immunoglobulin heavy chain enhancer [13]. Any twenty-five base pair sequence is very rare and this one does not occur exactly in our positive test set. If two mismatches are allowed then it appears twice–not surprisingly in/near the two S/MARs that gave rise to the sequence. The use of this sequence therefore depends entirely on how many mismatches are allowed, and since this sequence is nearly all A/T this method becomes simply a measure of AT-richness over two dozen bases and we have not pursued this particular approach further.

#### H-Rule

It has been found that a long run of bases that do not contain a G binds to the matrix [14]. MAR-Wiz [15] allows users to use this rule where it is translated into their formalism as a motif of 20 consecutive Hs (i.e. each of the 20 bases is an A, T or C) and allowing no mismatches. We call this MAR-Wiz implementation the H'-Rule. We have also implemented a version, which we call the H-Rule, which is a simple count of the number of occurrences (possibly overlapping) of a motif of 20 consecutive Hs on either strand in a moving window of 1000 bases, allowing two mismatches. We have quoted most of our results for this latter measure as it usually performs better, but as we discuss there are some interesting features of the H'-Rule.

#### MRS Signature

The two part motif AATAAYAA and AWWRTAANNWWGNNNC has been suggested by van Drunen *et al. *[12] to be an indicator of an S/MAR, where Y = C or T, W = A or T, R = A or G, and N = A or C or G or T.

These motifs should appear within about 200 bp of each other and can be on either strand, in either order and may even be overlapping. The 8 base part should match exactly and the 16 base part is allowed one base mismatch. The authors found that this signature identified 80% of S/MARs in their test set.

#### SMARTest

Frisch *et al. *[10] explain in general terms that SMARTest is based on 97 weight matrices, describing motifs of length 10 to 21 bases, which are AT-rich. These motifs were obtained by automatic searching in 34 known animal and plant S/MARs. Their testing on an independent test set gave comparatively good results finding 14 out of 37 known S/MARs and 19 out of 28 predictions to be correct–that is a sensitivity of 38% and precision of 68%, where sensitivity = TPTP+FN
 MathType@MTEF@5@5@+=feaafiart1ev1aaatCvAUfKttLearuWrP9MDH5MBPbIqV92AaeXatLxBI9gBaebbnrfifHhDYfgasaacH8akY=wiFfYdH8Gipec8Eeeu0xXdbba9frFj0=OqFfea0dXdd9vqai=hGuQ8kuc9pgc9s8qqaq=dirpe0xb9q8qiLsFr0=vr0=vr0dc8meaabaqaciaacaGaaeqabaqabeGadaaakeaadaWcaaqaaiabdsfaujabdcfaqbqaaiabdsfaujabdcfaqjabgUcaRiabdAeagjabd6eaobaaaaa@348C@, and precision (sometimes called specificity) = TPTP+FP
 MathType@MTEF@5@5@+=feaafiart1ev1aaatCvAUfKttLearuWrP9MDH5MBPbIqV92AaeXatLxBI9gBaebbnrfifHhDYfgasaacH8akY=wiFfYdH8Gipec8Eeeu0xXdbba9frFj0=OqFfea0dXdd9vqai=hGuQ8kuc9pgc9s8qqaq=dirpe0xb9q8qiLsFr0=vr0=vr0dc8meaabaqaciaacaGaaeqabaqabeGadaaakeaadaWcaaqaaiabdsfaujabdcfaqbqaaiabdsfaujabdcfaqjabgUcaRiabdAeagjabdcfaqbaaaaa@3490@, where TP = number of true positives, FP = number of false positives, FN = number of false negatives. Note that it was possible for more than one "prediction" to predict the same S/MAR. The same authors compared MAR-Finder on the same data and in their hands it found 12 out of 37 known S/MARs and 20 out of 25 predictions to be correct–that is a sensitivity of 32% and a precision of 80%. We used the SMARTest program as supplied on the public website.

#### ChrClass

We have used the package as supplied from the public website [16] and we have used the results for "predicted S/MARs" ignoring any output for the MRS and using the column "score" as a variable threshold in the ROC analyses. In the figures giving ROC curves, "C" marks the point where all the predictions of ChrClass for "predicted S/MARs" are included.

#### Duplex Destabilisation

SIDD calculations predict where the DNA strands can easily separate: it has been suggested that this is an indication of the presence of an S/MAR [19]. This is plausible both because DNA melting tends to occur in regions high in AT and there is some association of S/MARs with origins of replication [31,32].

These calculations assume that DNA is under torsional stress and it will relieve this stress by melting. Energy is needed to separate the strands but the energy needed to twist a pair of separated strands is less than the energy to twist the equivalent length of double stranded DNA. These facts form the basis of a thermodynamic model [19,33,34] which calculates the energies and probabilities of different states with different positions of base separation. One output is the p-graph which gives the probability that a given base pair is separated–typically this graph shows a peak in a small region and is close to zero elsewhere. The G-graph gives the average energy which has to be put into the system for a given base pair to separate and is normally a more sensitive measure: following the advice in the references this is the quantity we have used for the duplex destabilisation method. We were able to get results corresponding to the public website up to an (inconsequential) off-set of about 1.5 to 2.0 kcal between the website results and our calculations for a given sequence.

Details of the method are in the above references. In short, the calculations are extremely time consuming, and we have limited the analysis to thermodynamic states of one or two open windows, on the grounds that this gives a good approximation to the result given with more open windows. Otherwise we used the default parameters of the public website. The reported results are based on calculations on a sequence length of 10 kb: exploratory analyses with longer and shorter sequences give similar results (not shown).

As noted in the introduction, the authors of [2,19,21] present a number of ideas and results but do not claim to give a prediction algorithm. This implementation takes the obvious step of testing whether the G-graph crosses below a given threshold.

#### Thermodyn

Thermodyn is a calculation of the free energy of strand separation derived from summing the contributions of each doublet in a window to the thermodynamic quantities Δ*H *and Δ*S *[17]. Its use in the context of finding S/MARs comes from Kieffer *et al. *[18] where it was used to check for the plausibility of the S/MARs found in that experiment. The formulae are straightforward to program and we have tried Thermodyn as a predictor in our analyses. After some experimenting, we used a window size of 1000 bases.

#### AT-percentage

A simple measure of AT-percentage was also tried as a control: this was calculated as the proportion of bases that are A or T in a sliding window of 300 bases. This proportion was associated with the central base of the window.

The additional files contain our C++ code for calculating several of these measures [see Additional file 10].

### Test Sets and Analytical Procedure

#### Positive Test Set

To evaluate the S/MAR predictors we constructed a test set of known S/MARs. S/MARs are defined according to one of several experimental protocols. There is some controversy in the field as to the validity of the protocols [35] and the underlying biology is still being clarified [36]: we therefore give a short explanation of the operational definitions.

In most *in vitro selection protocols*, the nuclei are isolated from cells, and the nuclear scaffolds are fractionated from these nuclei with either LIS-containing buffer–that is a low salt extraction buffer containing lithium diiodosalicylate–or a high-salt buffer. The nuclear scaffolds are digested with restriction enzymes, incubated with labelled DNA fragments in the presence of some competitor DNA and centrifugated. The DNA is purified and analysed on agarose gel and via autoradiography [37]. The first steps in the usual *in vivo selection protocols *are the same, but after restriction digest, the nuclear scaffolds are directly centrifugated. The DNA is purified from both supernatant (non-S/MAR) and pellet (S/MAR) fraction and analysed on agarose gel and southern hybridisation [4,37], or directly cloned and sequenced [38]. For *UV-crosslinking *the cells are UV-radiated, and the nuclear lamina is purified. The DNA fragments covalently linked to the lamina proteins *in vivo *are cloned and sequenced [39]. In the *topoisomerase II cleavage assay*, an incubation with topoisomerase II is followed by Proteinase K digest, phenol-extraction and DNA purification. The analysis of the DNA fragments is done by gel electrophoresis, restriction digests, Southern transfer, hybridisation, and PCR amplification [40].

It can be seen that there are a number of ways in which subtypes of S/MARs might be defined according to the experimental definition. One division of the protocols is into *in vivo *protocols which find S/MARs as they occur in cells and *in vitro *protocols which test if known sequences of DNA can act as S/MARs. Another interesting division is between those protocols which use LIS and those that do not–the former is thought to be a more disruptive technique and hence finds fewer S/MARs.

We obtained our positive sequences from two sources, about two thirds from the S/MARt DB, which is built up from a literature search [41], and one third from the S/MARs found in the experiment of Purbowasito *et al. *[24] for one sequence of one megabase. In both cases, the defined mouse and human sequences were blasted against the mouse genome (asssembly build NCBIM33) or human genome (assembly build NCBI35) to find the surrounding chromosomal sequence. In essence the reported analyses refer to the S/MAR sequences themselves, but there are several reasons why the analyses need the surrounding sequence–for example many of the methods use moving windows of length 1 kb. As noted below we also credit the methods with near misses. To make sure these minor needs have been met, the analyses have used a sequence of 10 kb with the S/MAR in the middle. Knowing the surrounding sequence has also allowed a number of exploratory analyses of the 100 kb region. The additional material contains various tables giving the DNA sequence, reference identifier and further information about the data we used [see Additional files 11, 12, 13].

We used version 2.3 of the S/MARt DB. To improve the power of the analysis, this data was purified as follows. Twelve sequences with undefined bases in the S/MAR sequence or in the vicinity were removed from the test set. We checked the original literature and removed seven S/MARs where insufficient experimental evidence was given. We excluded S/MARs longer than 5000 bases. This produced a set of 113 known S/MARs (86 human and 27 mouse) which had been confirmed as follows:

16 by *in vivo *selection only [29,38,42-46],

17 by *in vivo *and *in vitro *selection [4,37,47-52],

1 by two different *in vivo *assays [45,53],

1 by *in vivo *selection [46] and FISH [3],

57 by *in vitro *selection only [14,37,54-69],

14 by UV-crosslinking [39],

6 by re-binding assays [18,70],

1 by topoisomerase-II cleavage assay [40].

113 S/MARt DB Total

The Purbowasito experiment [24] was based on an *in vitro *binding assay using high salt and this provided a further 52 S/MARs to make a total of 165 S/MARs. We also checked that no S/MAR was included twice in the dataset.

#### Control Test Sets

We evaluated the performance of the S/MAR predictors on the positive test set by comparing the performance against a control test set. We have four control datasets, which we call "background", "coding", "negative" and "*E. coli*". We regard the background test set as the most useful for this purpose: the choice of control dataset is discussed in the Discussion section.

Each control set consists of 330 sequences and was used in the same way. A sub-sequence in the middle of each sequence was imagined to be an S/MAR and part of the analysis was to see if the imaginary S/MAR was (wrongly) identified by the prediction methods. We call the imaginary S/MAR a pseudo S/MAR. The chance of finding the pseudo S/MAR increases with its length and to control for this we matched two sequences in the background set to one sequence in the positive test set and assumed that each of the pseudo S/MARs had the same length as the matched real S/MAR.

The *background *test set was assembled by selecting sequences at random from the mouse genome. If a sequence contained an undefined base, an N, then that sequence was discarded and another one chosen. The *coding *test set was prepared by concatenating coding sequences and then dividing the concatenation into long sections. The coding sequences were taken to be the exons of coding proteins, excluding the first and last exon, from ENSEMBL mouse assembly 33. The *negative *test set consists of artificial sequences derived from a 3rd order Markov model: that is each sequence was built by adding a base chosen at random based on the conditional probabilities of the three preceding bases. These probabilities were derived by sampling sequences from the whole of the mouse genome. The *E. coli *test set is a selection of non-overlapping sequences from the *E. coli *K12 substrain MG1655 taken from GenBank sequence reference U00096.

#### Analytical Procedure

The profile of each of the measures was calculated for each sequence in the test sets and an S/MAR is predicted where the measure shows a peak. However, the precise algorithm will contain parameters for the necessary height and width of the peak. The height or threshold can be varied to alter the balance between the proportion of S/MARs found and the false positive rate.

We also included two parameters to allow predictions to be successful for near misses. These parameters were set in the light of the following information. It is known that functional S/MARs can be very short and one set of experiments found there is no need to make S/MARs longer than 300 bases [71]. We gave statistics above on the length of S/MARs in the dataset. The authors of MAR-Finder advise that in their method there should be a significant value, for a sliding window of 1000 bases, at three consecutive positions 100 bases apart on the assumption that S/MARs are about 600 bases long.

The following procedure has been adopted. The measure had to exceed the threshold for at least a given number of bases, *x*. For MAR-Finder and the MAR-Finder version of the H-Rule *x *was taken to be 201 bases (for the reason explained above) and for all other methods *x *was taken to be 1. These islands were then extended on each side by *y *bases, *y *being chosen to make up the run to 600 bases, i.e. *y *was 200 for MAR-Finder and 300 for the other methods, *y *was put equal to 0 for Thermodyn and the H-Rule as this worked well for these methods. Given the lack of ambiguity for the ChrClass and SMARTest methods, we used the predictions as they stood–i.e. *x *is irrelevant, and *y *was taken as 10 as a protection against any difficulties in finding the S/MAR in the genome assembly.

Islands which were less than *z *bases apart were then merged: i.e. the region between the islands regarded as part of the larger S/MAR. *z *was taken as 100 bases. Any method of analysis will have implicit values for *x*, *y *and *z*. However, our exploratory analyses show that our conclusions are robust over a wide range of these parameters.

Finally the method is taken to have predicted a particular S/MAR or pseudo S/MAR if any one of its bases has been identified as part of an S/MAR by this procedure.

## Authors' contributions

GK suggested the topic and provided the early initiative. AH did the literature review and advised on experimental techniques. SO prepared the data. KE programmed and carried out the main and exploratory analyses–this included a reprogramming of the MAR-Finder method and SIDD calculations. SO and AH checked the programming and SO checked the analysis–much of it by reprogramming. KE wrote the drafts with comments from SO and AH. LW and GK gave technical advice and supervised the final editing. All authors discussed the implications of the results and read and approved the final draft.

## Supplementary Material

Additional File 1 Notes on the other additional files. Click here for file

Additional File 2 ROC Curves for the other control data sets. Click here for file

Additional File 3 S/MARs found at Table 1 thresholds. Click here for file

Additional File 4 Approximate thresholds for finding S/MARs. Click here for file

Additional File 5 Sequence data for the positive dataset. Click here for file

Additional File 6 Sequence data for background sequences. Click here for file

Additional File 7 Sequence data for coding sequences. Click here for file

Additional File 8 Sequence data for negative data set. Click here for file

Additional File 9 Sequence data for *E. coli*. Click here for file

Additional File 10 C++ code: a collection of files combined into one file using the unix .tar.gz format. See the README file for usuage. Click here for file

Additional File 11 Notes on the positive sequences part A1 (protocol etc.). Click here for file

Additional File 12 Notes on the positive sequences part A2 (exclusions). Click here for file

Additional File 13 Notes on the positive sequences part B (data from Purbowasito *et al*.). Click here for file
