# Supplementary material for: A comparative study of S/MAR prediction tools
Source: BMC Bioinformatics. 2007 Mar 2;8:71. doi: 10.1186/1471-2105-8-71 (PMC1847452; doi:10.1186/1471-2105-8-71)
Supplement: Additional File 2 — ROC Curves for the other control data sets. [file 1471-2105-8-71-S2.pdf]

Additional File 2—Graphs for the other control datasets

Explanation

In the text we explained that we preferred the use of the background dataset as the control dataset, but that we had performed the analyses using the coding, negative and *E. coli* datasets. This file gives the ROC curves for these analyses. The graphs correspond to the graphs in the main text.

Figures 1 to 10 use the coding dataset as control, Figures 11 to 20 and the negative dataset as control and Figures 21 to 30 the *E. coli* dataset as the control.

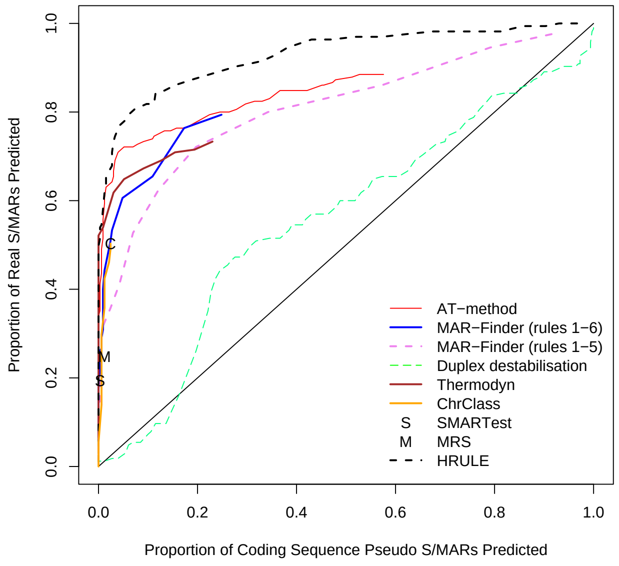

Figure 1: ROC curve for the positive dataset versus coding sequences

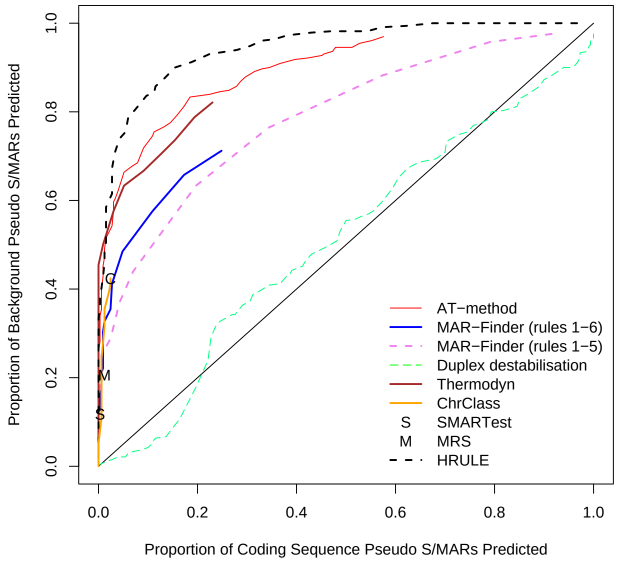

Figure 2: ROC curve for the background dataset (that is randomly selected real mouse DNA) versus coding sequences

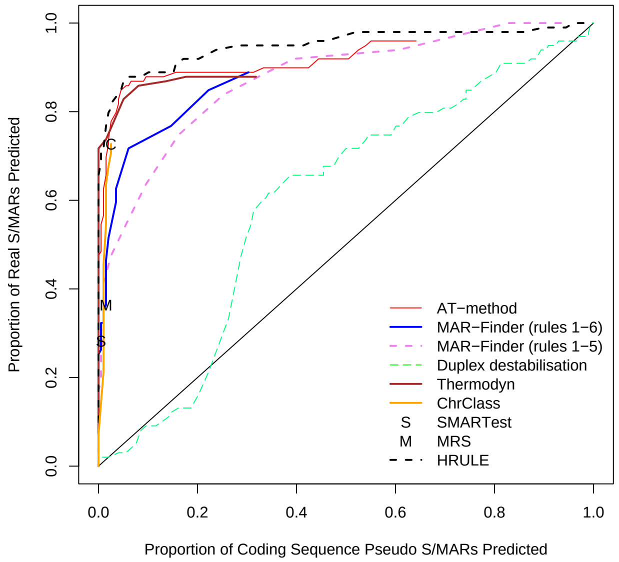

Figure 3: ROC curve for long positive S/MARs versus coding sequences

Figure 4: By the definitions used for the other datasets there are no AT-rich 10kb regions in the coding dataset and so the corresponding ROC curve is omitted.

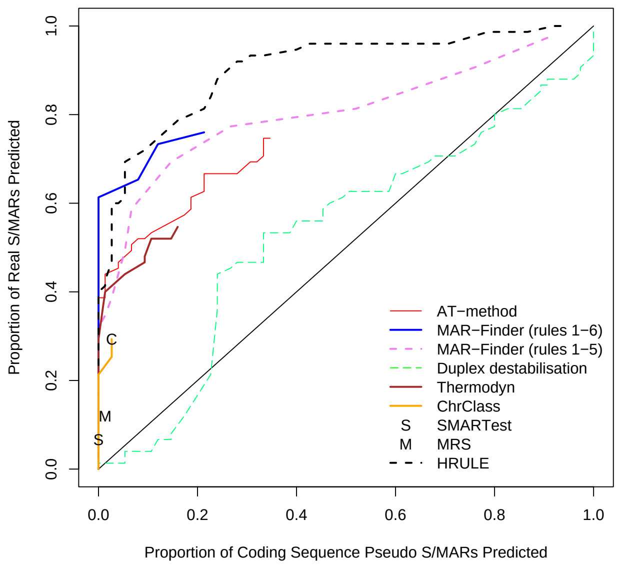

Figure 5: ROC curve for AT-poor 10kb regions versus coding sequences

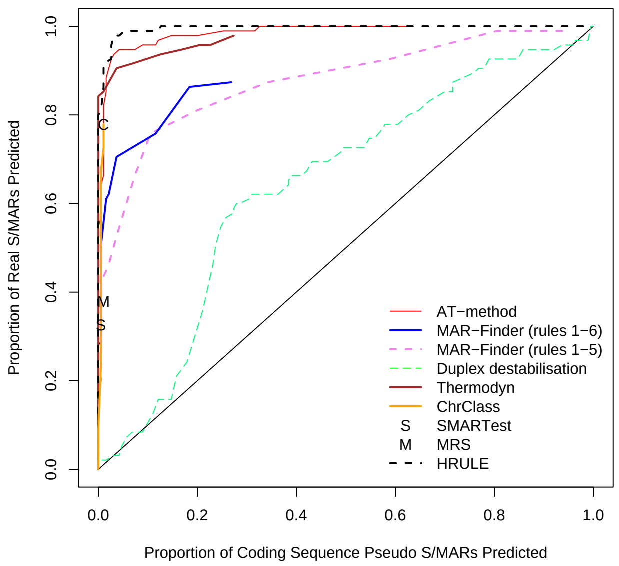

Figure 6: ROC curve for finding S/MARs that are AT-rich versus coding sequences

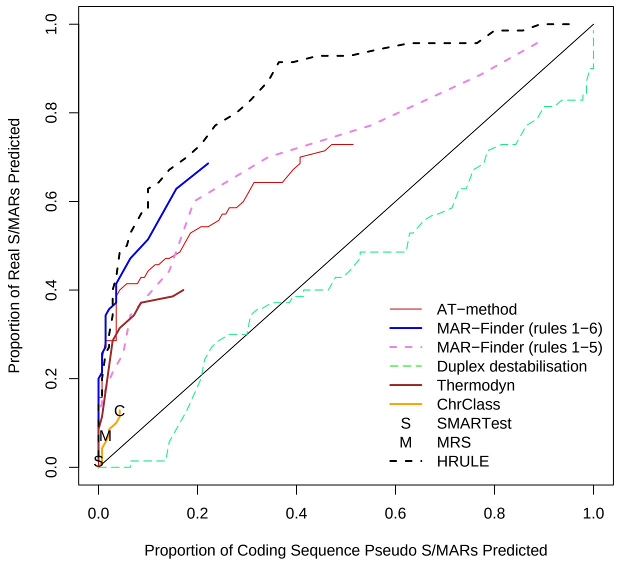

Figure 7: ROC curve for finding S/MARs that are AT-poor versus coding sequences

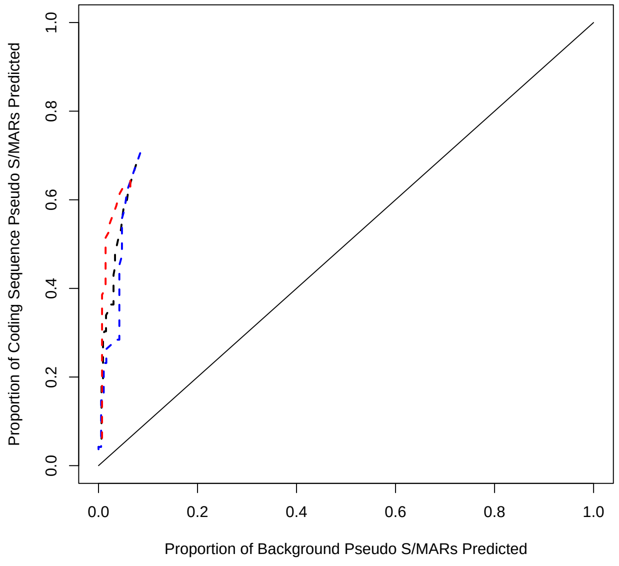

Figure 8: ROC curve for the H′-Rule for three divisions of the dataset — positive compared with coding sequences. a) black (middle curve) using all the positive dataset; b) red (top curve) AT-poor S/MARs; c) blue (bottom curve) AT-rich S/MARs.

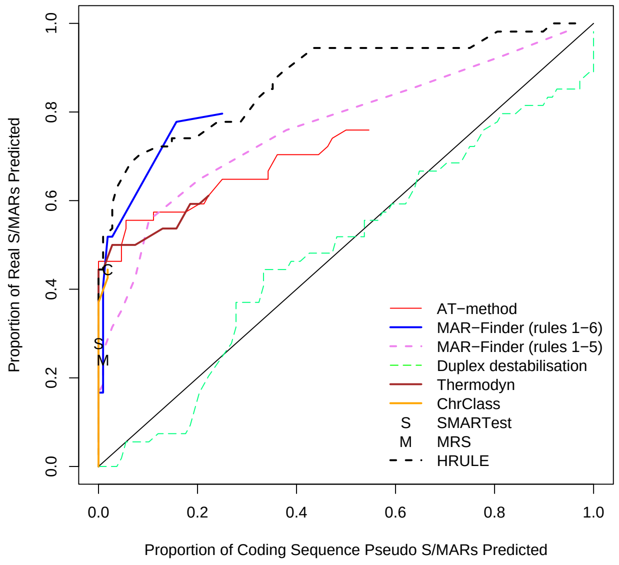

Figure 9: ROC curve for the positive with-LIS data versus coding sequences

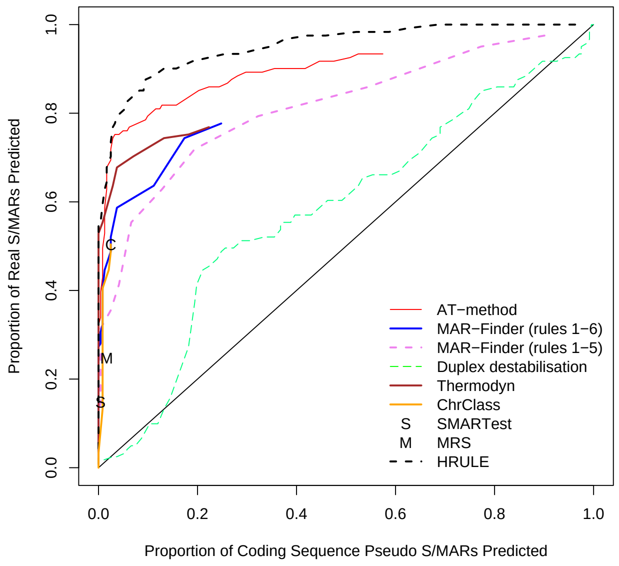

Figure 10: ROC curve for the positive non-LIS data versus coding sequences

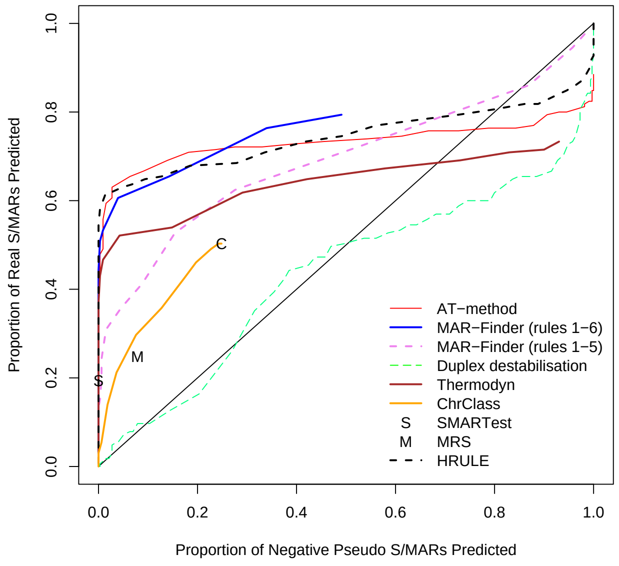

Figure 11: ROC curve for the positive dataset versus negative dataset

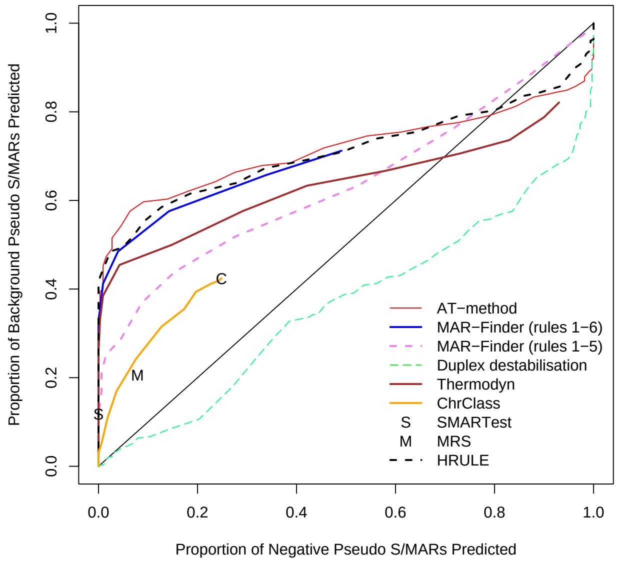

Figure 12: ROC curve for the background dataset (that is randomly selected real mouse DNA) versus negative dataset

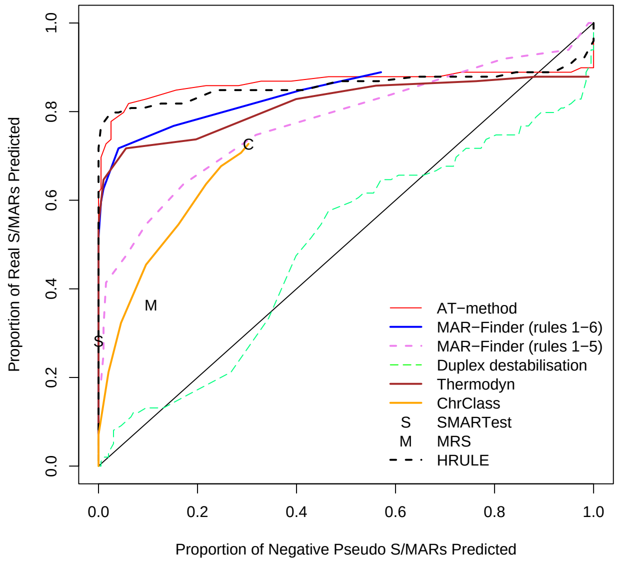

Figure 13: ROC curve for long positive S/MARs versus negative dataset

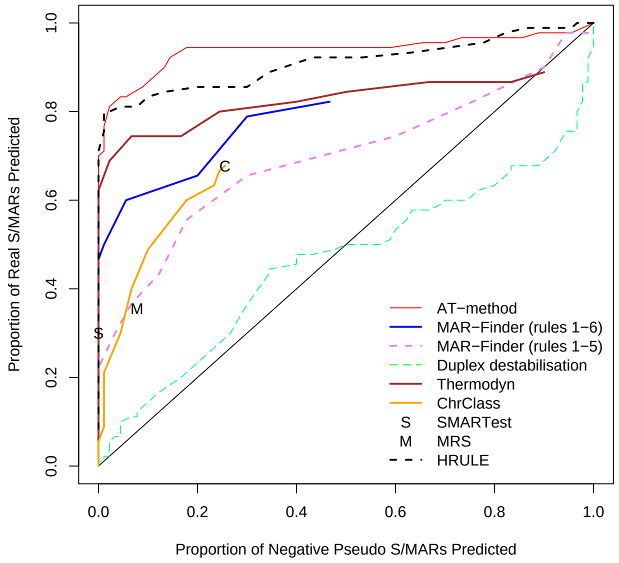

Figure 14: ROC curve for AT-rich 10kb regions versus negative dataset

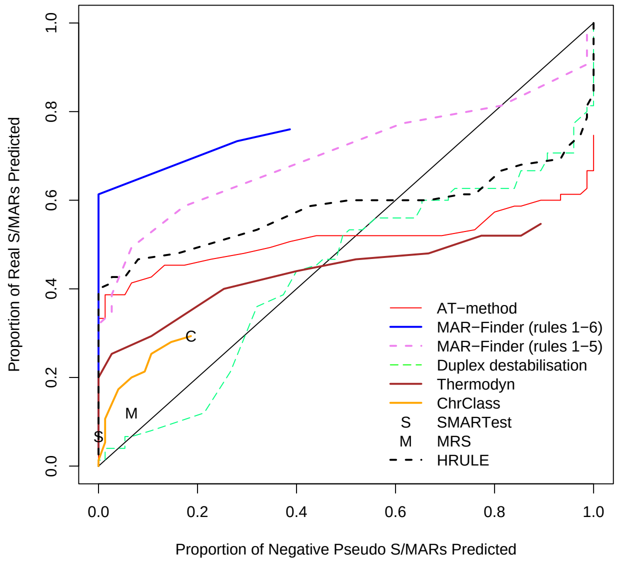

Figure 15: ROC curve for AT-poor 10kb regions versus negative dataset

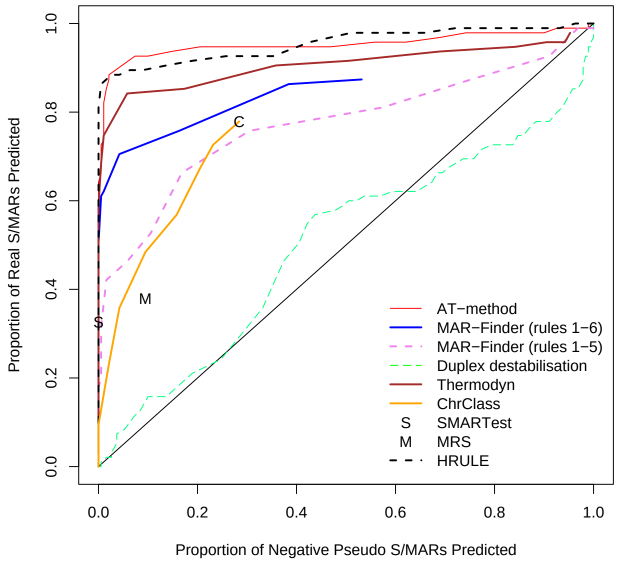

Figure 16: ROC curve for finding S/MARs that are AT-rich versus negative dataset

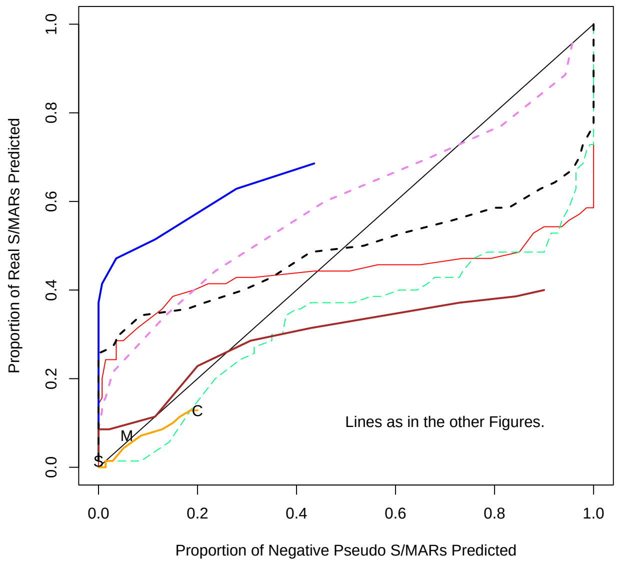

Figure 17: ROC curve for finding S/MARs that are AT-poor versus negative dataset

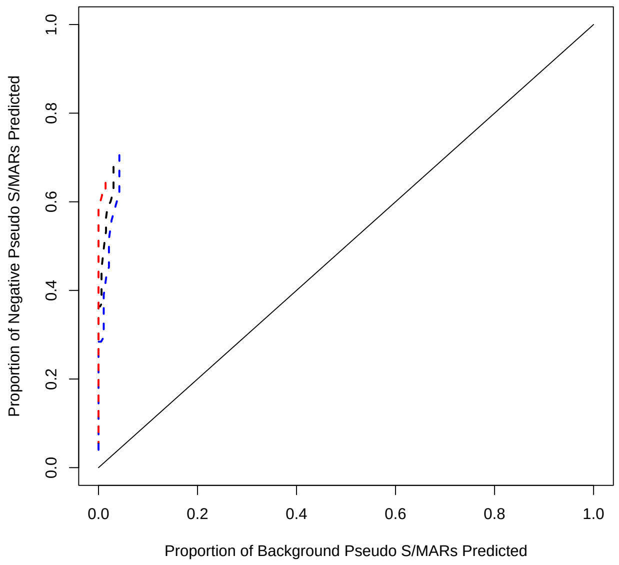

Figure 18: ROC curve for the H′-Rule for three divisions of the dataset — positive compared with negative dataset. a) black (middle curve) using all the positive dataset; b) red (top curve) AT-poor S/MARs; c) blue (bottom curve) AT-rich S/MARs.

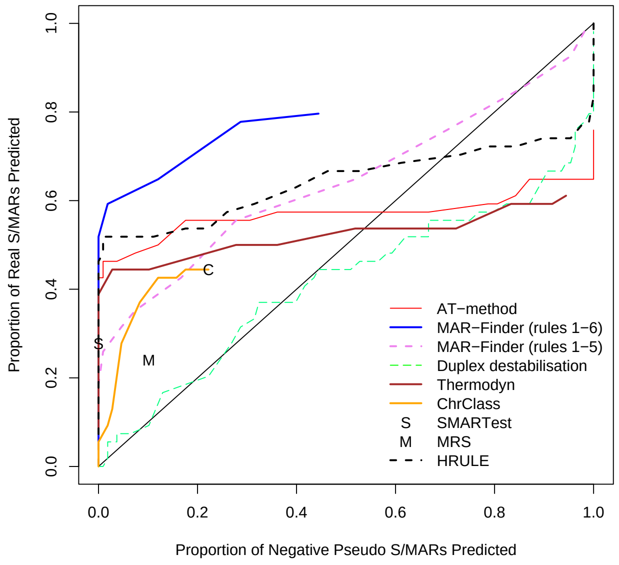

Figure 19: ROC curve for the positive with-LIS data versus negative dataset

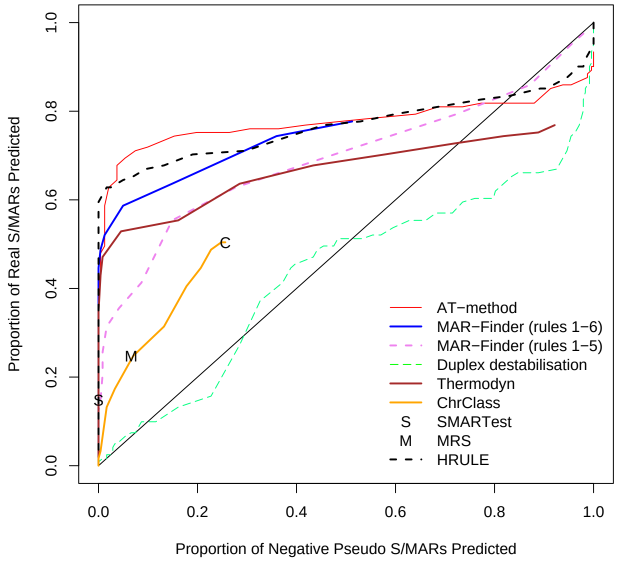

Figure 20: ROC curve for the positive non-LIS data versus negative dataset

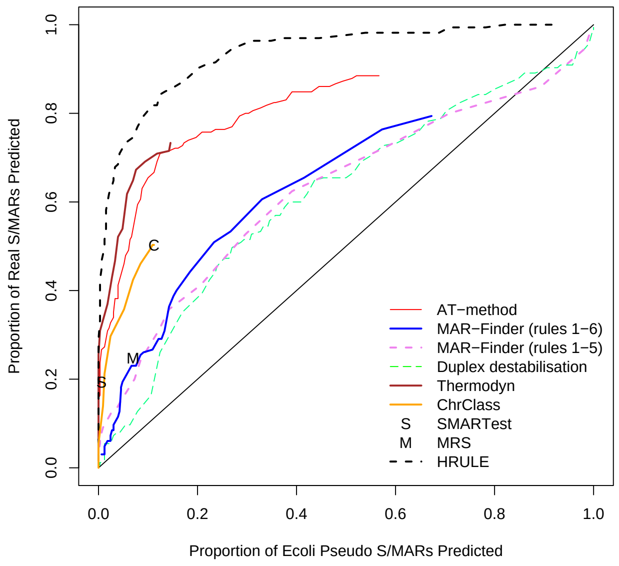

Figure 21: ROC curve for the positive dataset versus *E. coli* dataset

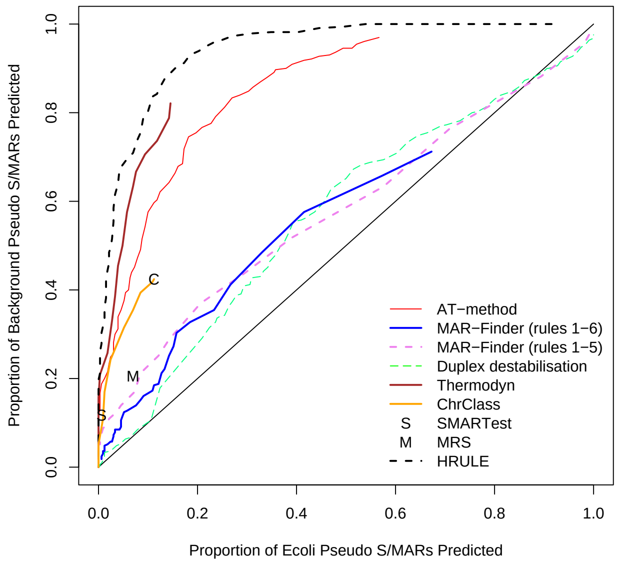

Figure 22: ROC curve for the background dataset (that is randomly selected real mouse DNA) versus *E. coli* dataset

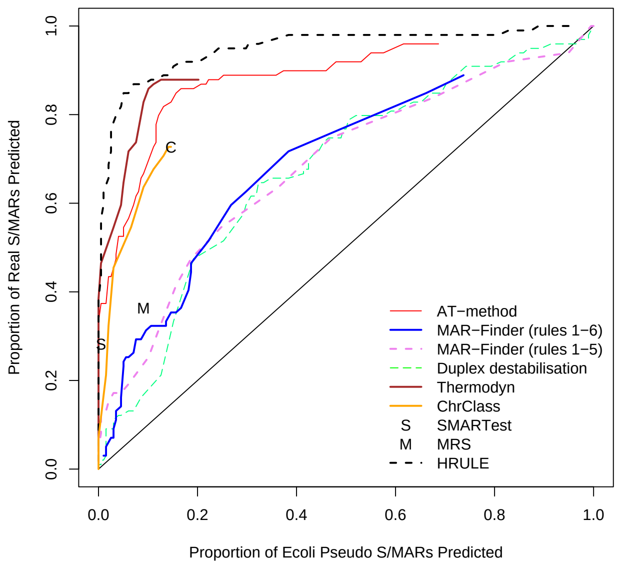

Figure 23: ROC curve for long positive S/MARs versus *E. coli* dataset

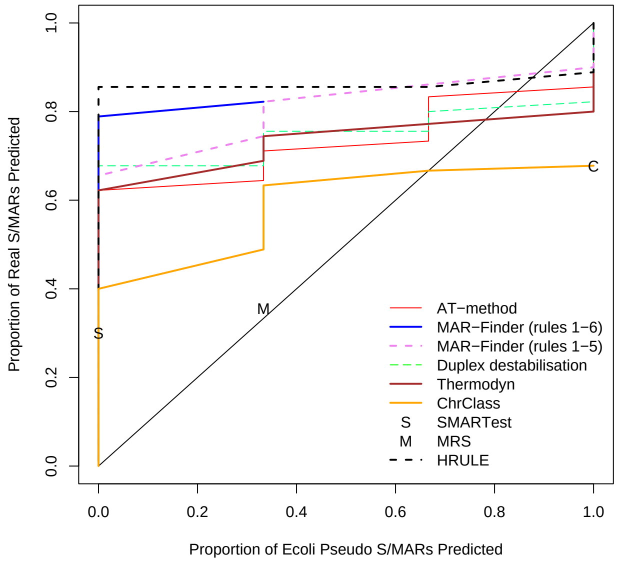

Figure 24: ROC curve for AT-rich 10kb regions versus *E. coli* dataset

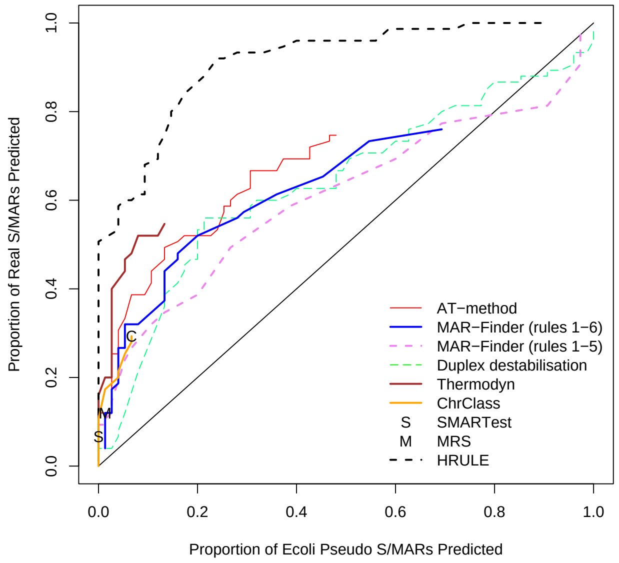

Figure 25: ROC curve for AT-poor 10kb regions versus *E. coli* dataset

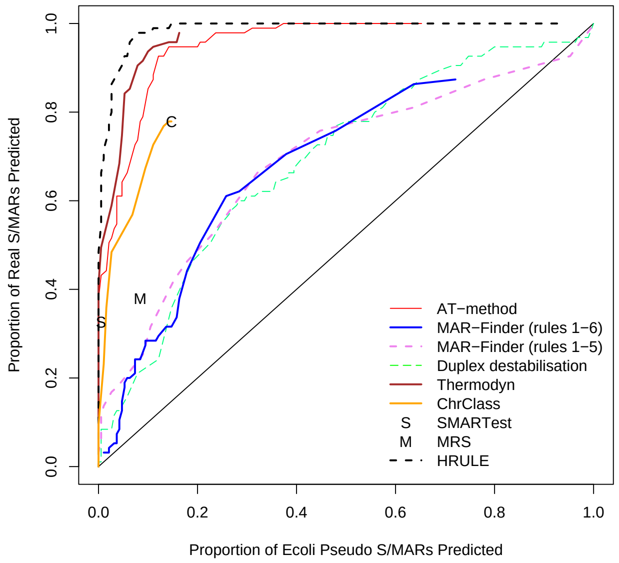

Figure 26: ROC curve for finding S/MARs that are AT-rich versus *E. coli* dataset

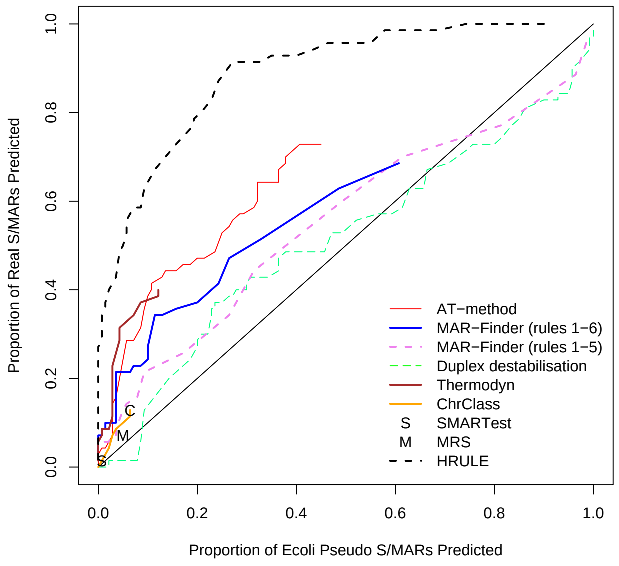

Figure 27: ROC curve for finding S/MARs that are AT-poor versus *E. coli* dataset

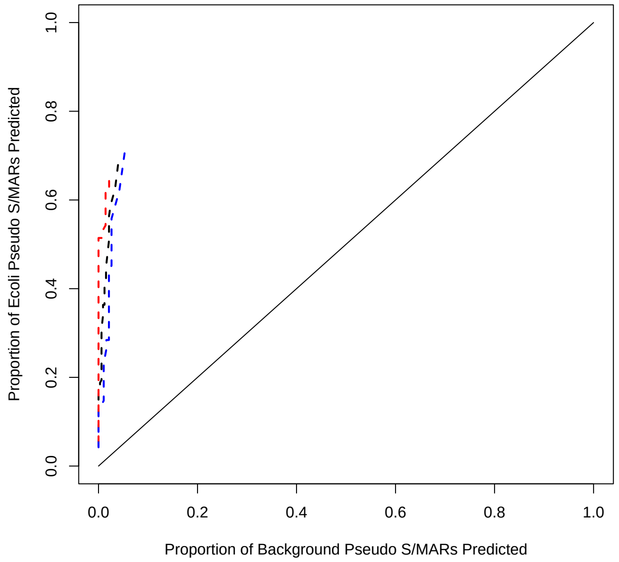

Figure 28: ROC curve for the H′-Rule for three divisions of the dataset — positive compared with *E. coli* dataset. a) black (middle curve) using all the positive dataset; b) red (top curve) AT-poor S/MARs; c) blue (bottom curve) AT-rich S/MARs.

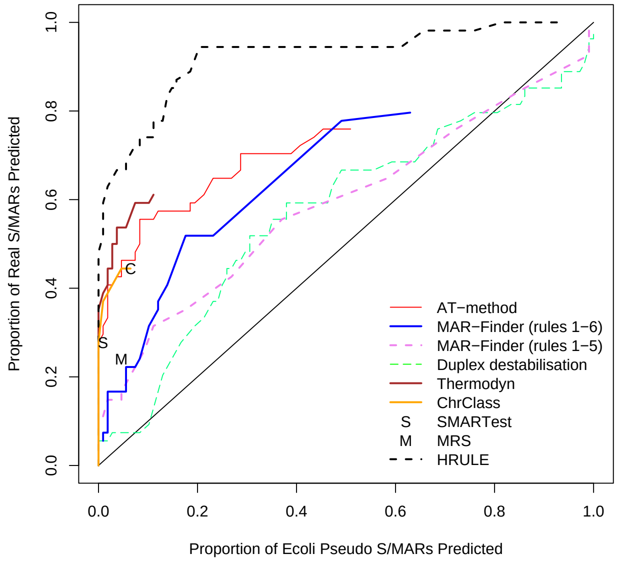

Figure 29: ROC curve for the positive with-LIS data versus *E. coli* dataset

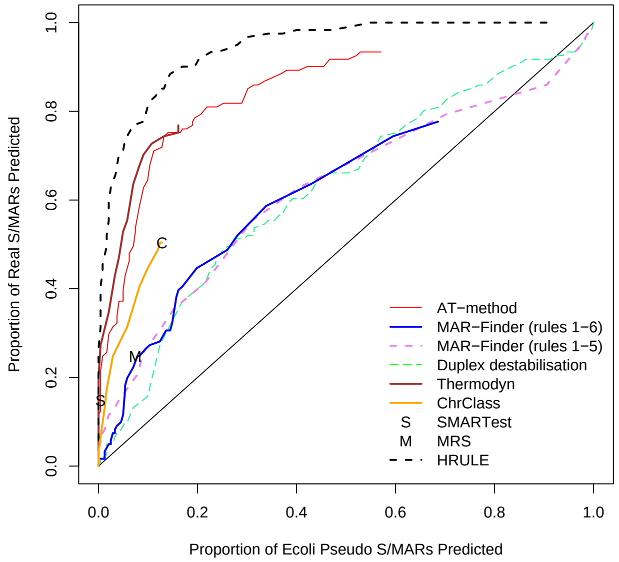

Figure 30: ROC curve for the positive non-LIS data versus *E. coli* dataset
